# A HER2-targeting antibody-MMAE conjugate RC48 sensitizes immunotherapy in HER2-positive colon cancer by triggering the cGAS-STING pathway

**DOI:** 10.1038/s41419-023-06073-8

**Published:** 2023-08-24

**Authors:** Xiaohan Wu, Lingyan Xu, Xiaofei Li, Yirui Zhou, Xiao Han, Wei Zhang, Weicheng Wang, Wenjie Guo, Wen Liu, Qiang Xu, Yanhong Gu

**Affiliations:** 1 Department of Oncology and Cancer Rehabilitation Centre, The First Affiliated Hospital of Nanjing Medical University, Nanjing, Jiangsu China; 2 State Key Laboratory of Pharmaceutical Biotechnology, Engineering Research Center of Protein and Peptide Medicine, School of Life Sciences, Nanjing University, Nanjing, China

**Keywords:** Targeted therapies, Pattern recognition receptors

## Abstract

Human epidermal growth factor receptor 2 (HER2) is a protein that is overexpressed in some types of cancer, including breast and urothelial cancer. Here we found that HER2 was present in a portion of colon cancer patients, raising the possibility of using anti-HER2 therapy. RC48, a novel antibody-drug conjugate (ADC) comprising cytotoxic monomethyl auristatin E (MMAE) and an anti-HER2 antibody tethered via a linker, showed a comparable therapeutic effect in both HER2 low expressed (IHC2+/FISH- or IHC+) and high expressed urothelial cancer patients. In vitro studies using colon cancer cell lines showed that RC48 effectively impeded the proliferation of HER2-positive cells, indicating its potential as a treatment for HER2-positive colon cancer. Mechanism study showed that RC48 not only induces cell cycle arrest but also disrupts HER2-mediated restain of cGAS-STING signaling, potentially activating an immune response against the cancer cells. The administration of RC48 significantly reduced the growth of HER2-positive colon cancer and made HER2-positive colon cancer cells more susceptible to immunotherapy. The results of our study will contribute to determining the feasibility of RC48 as a therapeutic option for HER2-positive colon cancer.

## Introduction

In 2022, colorectal cancer (CRC) is the second most commonly diagnosed type of cancer there were estimated 592,232 new cases, and the fifth leading cause of cancer death in China [1]. The standard therapy for advanced or metastatic CRC is chemotherapy that includes oxaliplatin or irinotecan combined with molecular-targeted agents that inhibit vascular endothelial growth factor (VEGF, e.g. bevacizumab) or epidermal growth factor receptor (EGFR, e.g. cetuximab). However, the response is unsatisfactory as the median overall survival (OS) is only around 30 months [2].

HER2, also known as ERBB2, is a member of the ERBB family of receptor tyrosine kinases (RTKs), including EGFR (ERBB1), HER2 (ERBB2), HER3 (ERBB3), and HER4 (ERBB4) [3]. These members play important roles in physiology and oncogenesis through multiple signaling pathways, such as the RAS/RAF/MEK/ERK and PI3K/AKT/mTOR pathways. HER2 alterations (amplification or mutations) are closely associated with various types of malignancies [4]. The frequency of HER2 expression in CRC varies in different studies due to measurement instability, such as immunohistochemistry. For instance, a study from 2017 reported that 1.4% of 4913 Chinese CRC patients were HER2-positive [5], while up to 40% of 170 CRC patients were positive in a report from 2004 [6]. However, it is generally accepted that HER2-positive patients account for about 7% [7–9]. Because overexpression of HER2 on the membrane can cause the homodimerization of HER2 or its combination with other ERBB family members to achieve effects similar to EGFR activation, HER2-positive patients have a high incidence of resistance to cetuximab, a commonly used EGFR-targeted agent in RAS wild-type mCRC [7, 10, 11]. As a result, inhibiting EGFR with cetuximab alone is insufficient. Therefore, therapies targeting HER2 are crucial for mCRC patients who have limited options due to their HER2-positive status.

Recent advancements in antitumor drugs have brought many HER2-targeting therapies, including single-epitope monoclonal antibodies (mAbs), small molecule tyrosine kinases inhibitors, and bispecific antibodies [3]. The development of mAbs has revolutionized cancer therapy by precisely targeting tumor surface antigens. However, treatment using mAbs alone is often inadequate due to its lower lethality against cancer cells compared to chemotherapy. Currently, the clinical therapies for CRC patients are mainly based on the combination strategy, such as trastuzumab plus pertuzumab with an overall response rate (ORR) of 32% [12], or trastuzumab plus the TKI inhibitor lapatinib with an ORR of 28% [13]. A new type of drug, antibody-drug conjugate (ADC) drugs, has been designed to make up for the limitations of HER2-targeted therapies. ADC drugs are monoclonal antibodies loaded with a small toxin molecule that can specifically target cancer cells and then produce a potent toxic effect [14].

Disitamab Vedotin (also known as RC48) is a new ADC drug comprised of Hertuzumab (also known as Disitamab) coupling with monomethyl auristatin E (MMAE) via a cleavable linker [15]. Despite being a recent addition to the market, it has achieved outstanding results in several trials of many malignancies. For instance, phase II clinical studies of HER2-positive gastric and urothelial cancer reported an ORR of 24.4% and 51.2%, respectively [16, 17]. Due to its impressive results, RC48 was granted breakthrough therapy status for HER2-positive urothelial cancer by the United States Food and Drug Administration (FDA) [15]. Interestingly, the clinical trials also showed that the response of patients with HER2 low expression (IHC2+/FISH- or IHC+) was similar to that of patients with HER2 high expression [18]. This is particularly significant since the majority of HER2-positive CRC patients have low HER2 expression, raising the question of whether RC48 would be effective in this type of cancer.

In this study, we evaluated the impact of RC48 on HER2-positive colon cancer cells both in vitro and in vivo and discovered that RC48 was able to significantly suppress HER2-positive colon cancer and enhance the effectiveness of anti-PD-1 therapy by activating the cGAS-STING pathway.

## Results

### HER2 was expressed in a subset of colon cancer

**Fig. 1:**
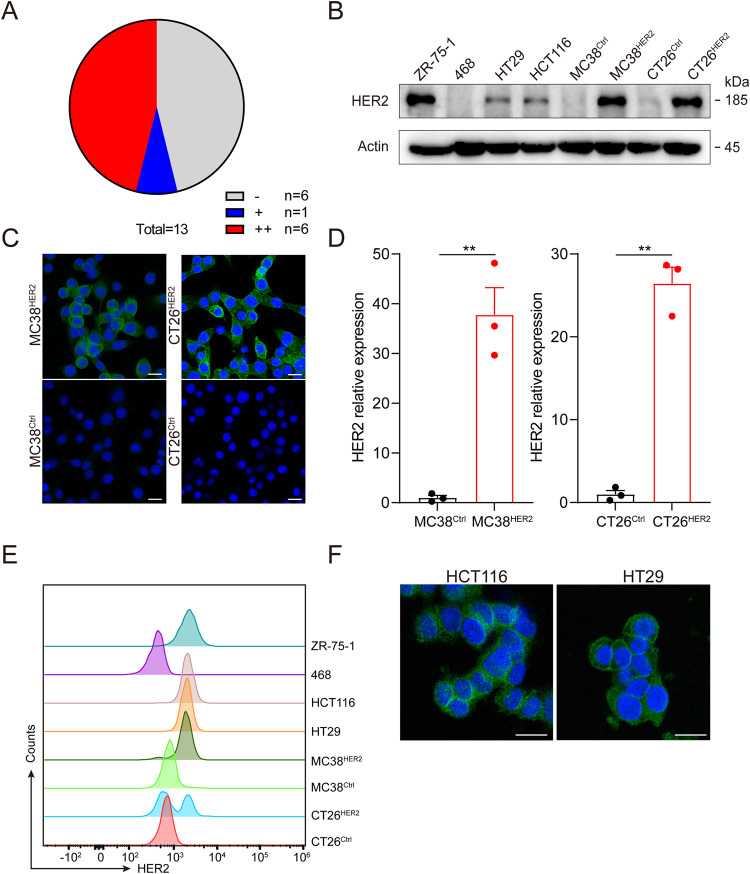
HER2 expression in colon cancer patients and cell lines. **A** Analysis of the immunohistochemical pathological reports for HER2 of 13 CRC patients in Jiangsu Provincial Hospital. **B** Western blot analysis of HER2 expression in murine and human CRC cell lines. **C** Expression and localization of HER2 detected by immunofluorescence on MC38^HER2^, MC38^Ctrl^, CT26^HER2^, and CT26^Ctrl^ cells. **D** The mRNA expression of HER2 was measured by qPCR. **P < 0.01. **E** The expressions of HER2 on the cell membrane were detected using flow cytometry. **F** The expression and localization of HER2 in HCT116 and HT29 cells were determined by immunofluorescence. Scale bars, 10 μm.

To assess the viability of the study and identify appropriate cells, we initially examined the HER2 expression in both the population and cell lines. Firstly, we conducted a review of the immunohistochemical results of patients from our hospital. Out of the 13 patients who underwent the HER2 test, 53.8% were found to be HER2-positive, with 7.7% showing HER2+ expression, 46.2% showing HER2 2+ expression, and 0% showing HER2 3+ expression (Fig. 1A). Next, we assessed HER2 expression in human and murine colon cancer cell lines through western blot analysis. The human breast cancer cell line ZR-75-1 was used as a positive control, while 468 was used as a negative control (Fig. 1B). The result showed that the HER2 expression levels in HCT116 and HT29 ranged between 468 and ZR-75-1. HER2-overexpressed cell lines (MC38^HER2^, CT26^HER2^) were generated using lentivirus transfection and HER2 was successfully overexpressed and located on the cell membrane, compared to MC38^Ctrl^ and CT26^Ctrl^ (Fig. 1C–E). The same location was also observed in HCT116 and HT29 cells (Fig. 1F). Besides, we conducted a FISH assay on HT29 and HCT116 cell lines and observed comparable quantities of CSP17 and HER2, suggesting the absence of HER2 amplification in these cells (Supplementary Fig. A). Though they are not amplified, HCT116 and HT29 are still suitable for further study because the only necessary condition for using RC48 is the expression of HER2.

### RC48 inhibited the growth of colon cancer cells in vitro

**Fig. 2:**
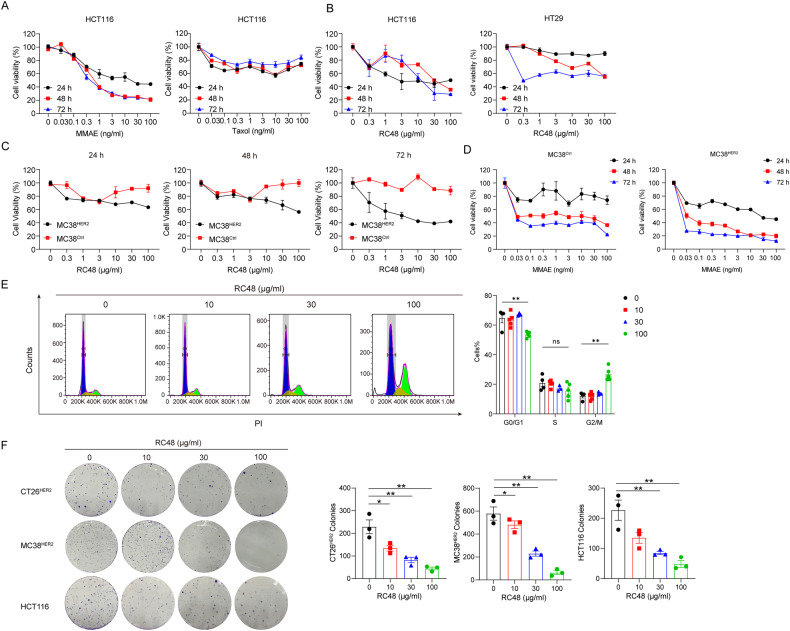
RC48 inhibited the growth of HER2-positive colorectal cancer cells in vitro. **A** In vitro growth inhibition assay for HCT116 cells treated with MMAE and Paclitaxel for 24 h. **B** In vitro growth inhibition assay for HCT116 and HT29 cells. **C** In vitro growth inhibition assay for MC38^HER2^ and MC38^Ctrl^ cells treated with RC48 for 48 h. **D** In vitro growth inhibition assay for MC38^Ctrl^ and MC38^HER2^ cells treated with MMAE for 24 h. **E** Flow cytometry analysis of cell cycle after treatment with different concentrations of RC48 for 48 h. **F** Clonogenic assay of HCT116, CT26^HER2^ and MC38^HER2^ treated with RC48. Data represent the mean ± SEM of three replicates. *P < 0.05, **P < 0.01, versus as indicated. n.s. not significant.

After confirming that the above cell lines were HER2 positive, we used them to determine the proliferation inhibition effect of RC48 in vitro. But the small molecule toxin component of RC48, MMAE, belongs to tubulin inhibitors, which are not commonly utilized in routine chemotherapy for CRC due to their lack of efficacy in clinical trials, such as paclitaxel. Thus, we first investigated the effect of MMAE on CRC cells. In in vitro growth inhibition assays, HCT116 was resistant to paclitaxel but still responsive to MMAE (Fig. 2A), indicating that RC48 treatment effectively suppressed the proliferation of CRC cells. Moreover, HCT116 showed a greater response to RC48 than HT29 (Fig. 2B). To determine the suitability of the HER2 overexpression murine cell line MC38^HER2^ for further research, we conducted similar experiments and found that RC48 selectively inhibited MC38^HER2^ compared to MC38^Ctrl^ (Fig. 2C), while MMAE showed the same inhibitory effect on both cell lines (Fig. 2D). These results demonstrate that RC48 selectively targeted HER2-positive cancer cells, which are dependent on the Disitamab. Besides, we found that the Disitamab alone did not have a growth inhibition effect (Supplementary Fig. D). Cell cycle analysis showed that RC48 arrested cells in the G2/M phase (Fig. 2E). Moreover, RC48 significantly inhibited the colony formation ability of colon cancer cells (Fig. 2F). These findings suggest that RC48 has an inhibitory effect on the HER2-positive colorectal cancer cells in vitro.

### RC48 triggered cGAS-STING activation in colon cancer cells

**Fig. 3:**
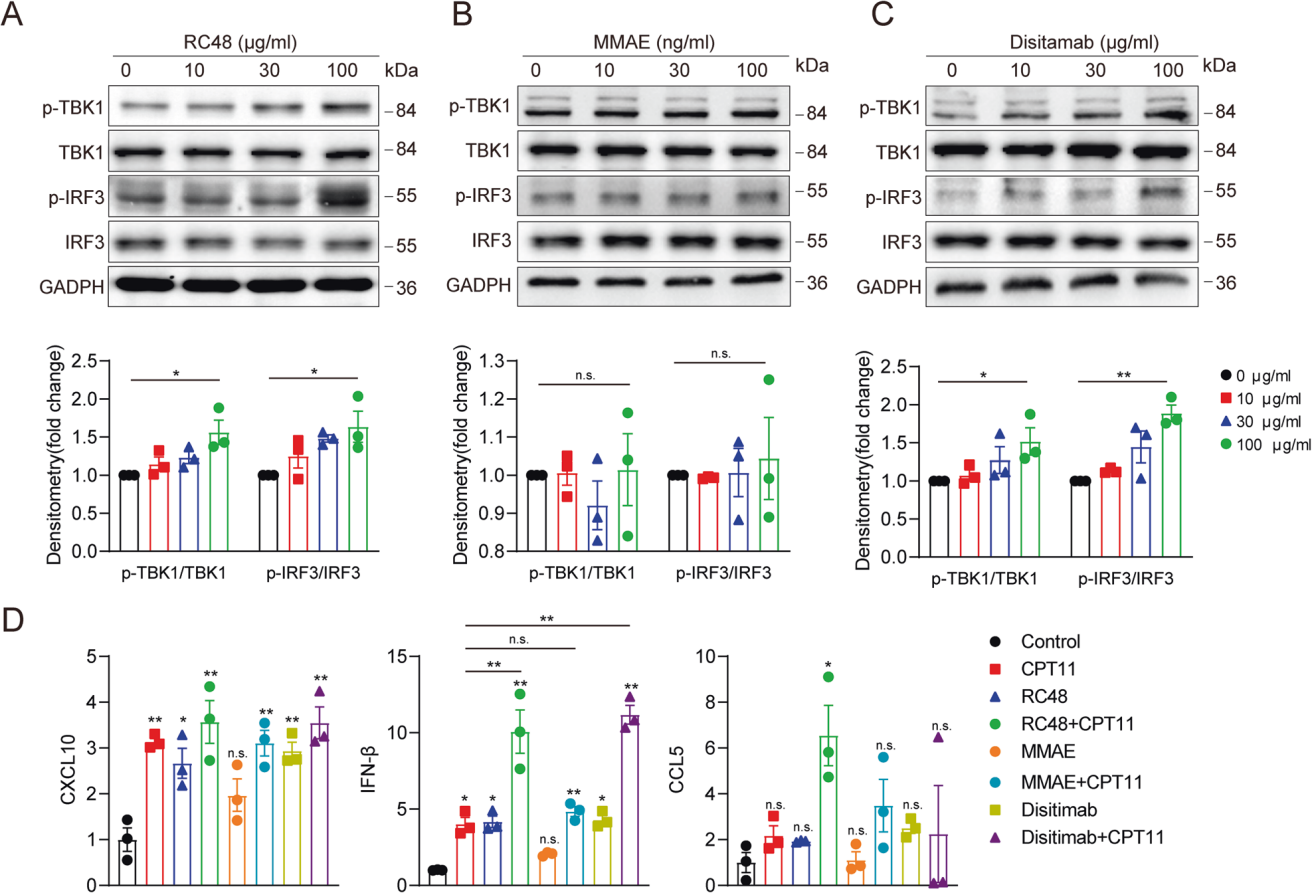
RC48 triggered cGAS-STING activation in colon cancer cells. **A**–**C** HCT116 cells were treated with different concentrations of RC48, MMAE, or Disitamab (0, 10, 30, 100 μg/ml) for 48 h, and the expression of p-TBK1, p-IRF3, TBK1, IRF3 protein was detected by immunoblotting. **D** The expression of the IRF3-responsive genes, CXCL10, IFN-β, and IFIT1 in HCT116 treated with 100 μg/ml RC48 for 48 h and 30 μM CPT11 for 12 h are detected by qPCR. *P < 0.05, **P < 0.01, versus as indicated. n.s. not significant.

To further explore the possible effect of RC48, we searched literatures related to tubulin inhibitors and found a previous study reporting that paclitaxel, a tubulin inhibitor like MMAE, could activate the cGAS-STING signaling pathway [19]. Based on that, we also found that RC48 could trigger the phosphorylation of IRF3 and TBK1 (Fig. 3A). To determine which component of RC48 was crucial for this activation, we applied the small molecule toxin, MMAE, and the monoclonal antibody, Disitamab, separately. The results showed that Disitamab alone generated the same effect as RC48, while MMAE did not (Fig. 3B, C). CPT11 was discovered to active cGAS-STING pathway in our previous study [20, 21]. The combination of RC48 and Disitamab increased the transcription of IRF3 responsive genes, IFN-β and CXCL10, and the upregulation in CCL5 was observed only in RC48 plus CPT11 group. Furthermore, the transcription of IFN-β was significantly elevated when RC48 and Disitamab were combined with CPT11. The effect was not observed with MMAE and was only significant when combined with CPT11 compared to control, but not with CPT11 alone (Fig. 3D). Our research initially focused on MMAE, but we unexpectedly found that the cGAS-STING could be activated by an anti-HER2 monoclonal antibody, leading us to explore the relationship between HER2 and the cGAS-STING pathway.

### RC48 released HER2-mediated cGAS-STING inactivation

**Fig. 4:**
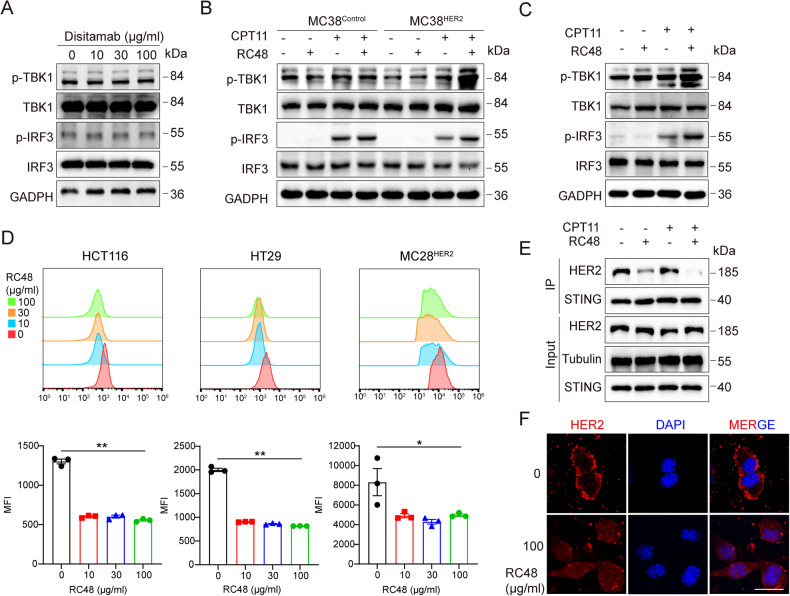
RC48 released HER2-mediated cGAS-STING inactivation. **A** MC38^Ctrl^ cells were treated with different concentrations of Disitamab (0, 10, 30, 100 μg/ml) for 48 h, and the expression of p-TBK1, p-IRF3, TBK1, IRF3 protein was detected by immunoblotting. **B** MC38^HER2^ and MC38^Ctrl^ cells were treated with 100 μg/ml RC48 for 48 h and 30 μM CPT11 for 12 h and the expression of p-TBK1, p-IRF3, TBK1, IRF3 protein was detected by immunoblotting. **C** HCT116 was treated with 100 μg/ml RC48 for 48 h and 30 μM CPT11 for 12 h. **D** The expressions of HER2 on the cell membrane were detected using flow cytometry. MFI Mean fluorescence intensity. **E** Co-immunoprecipitation of endogenous HER2 and STING in HCT116. **F** The expression and localization of HER2 in MC38^HER2^ were detected by immunofluorescence. *P < 0.05, **P < 0.01 versus as indicated. n.s. not significant. Scale bar, 10 μm.

Through literature search, we found that HER2 could interact with STING and inhibit the activation of its downstream signaling pathway [22]. The failure of RC48 to trigger the phosphorylation of IRF3 and TBK1 in MC38^Ctrl^ cells confirmed the requirement of HER2 for the activation of the cGAS-STING pathway (Fig. 4A). We observed that the phosphorylation levels of IRF3 and TBK1 triggered by CPT11 decreased when HER2 was overexpressed, but this was restored after RC48 treatment (Fig. 4B). Similar results were obtained by western blotting on HCT116 cells (Fig. 4C). To further determine whether this effect was due to the interaction of HER2 and STING reported in the previous literature, we conducted microscopy and FACS experiments observing that HER2 on the membrane will internalize after stimulated by RC48 (Fig. 4D, F). Then, we conducted a co-immunoprecipitation experiment and found out that the binding of HER2 and STING was significantly inhibited after the addition of RC48 (Fig. 4E). These results indicate that HER2 on the cell membrane will be internalized after being stimulated by RC48. According to the principle of RC48, internalized HER2 will enter lysosomes for degradation. Even if it is not degraded, the occupancy of RC48 reduces the amount of HER2 that can bind to STING. Therefore, the combination between them will weaken and cGAS-STING will relatively be activated.

### The combination of RC48 and anti-PD-1 significantly inhibited tumor growth in the syngeneic model of CRC

**Fig. 5:**
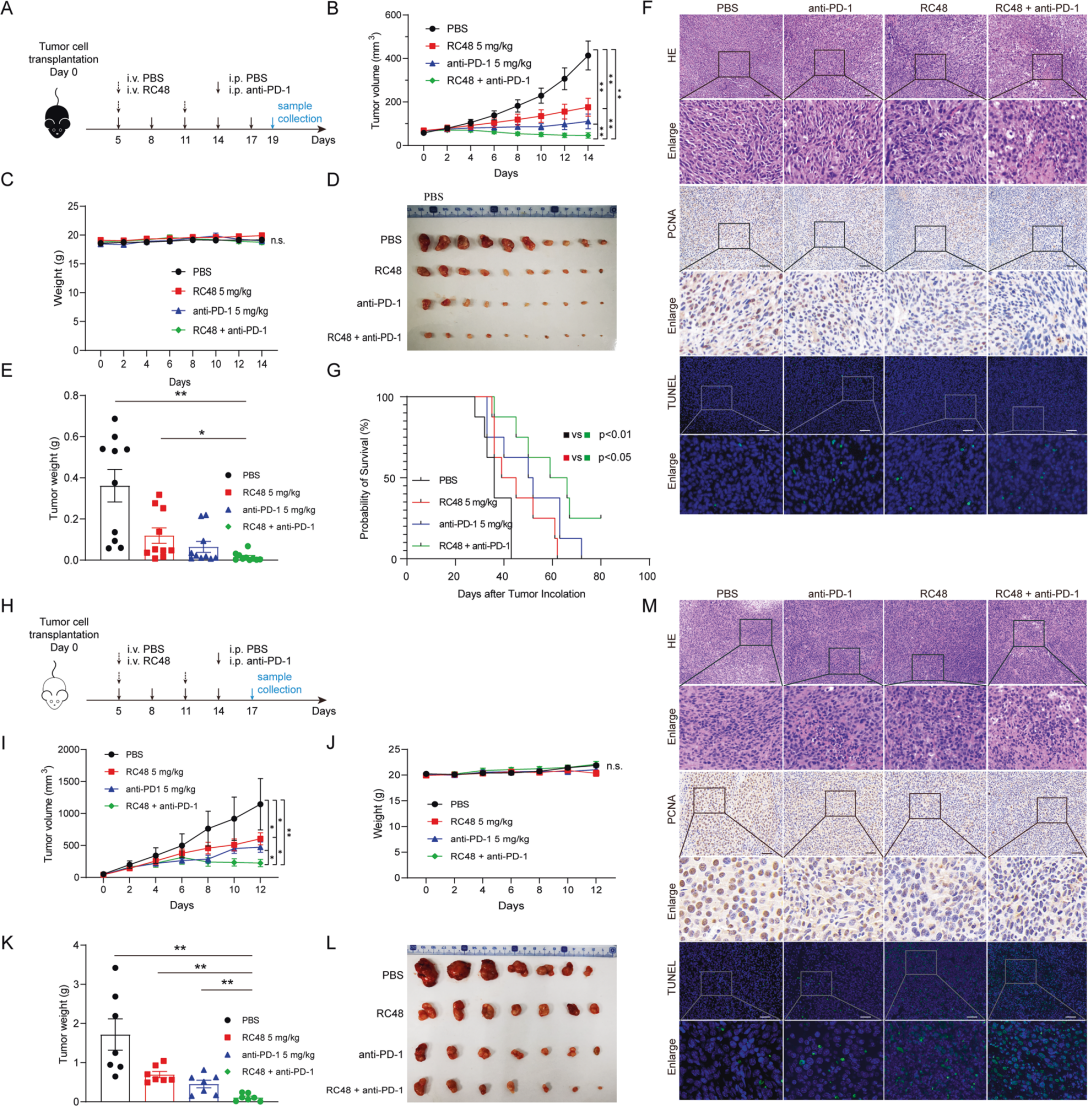
Combination of RC48 and anti-PD-1 significantly inhibited tumor growth in the syngeneic model of CRC. **A**–**E** MC38^HER2^ (1 × 10^6^) cells were inoculated in the C57BL/6 mice. Drugs were given as described in Materials and Methods. **B**, **C** Tumor volume and body weight were measured every two days. **D**, **E** Solid tumors were separated and weighed after the mice were sacrificed. The data represent the mean ± SEM of 10 mice per group. **F** Paraffin sections of tumor tissues were analyzed by H&E staining, PCNA immunohistochemistry staining, and TUNEL fluorescent staining. **G** The survival of tumor-bearing mice. **H**–**M** Same experiment with CT26^HER2^ in BALB/c mice. The data represent the mean ± SEM of 7 mice per group. *P < 0.05, **P < 0.01 versus as indicated. n.s. not significant. Scale bar, 50 μm.

We have already known that RC48 was capable of activating the cGAS-STING signaling pathway, which is important for antitumor immunity. Also, the combination of anti-HER2 therapy and immunotherapy has shown promising results in treating various cancers in clinical studies [23]. In addition, research has shown that RC48 could enhance the efficacy of immunotherapy in breast cancer [24]. Considering the above factors, we decided to explore the relationship between RC48 and immunotherapy in CRC. So, we established a murine syngeneic tumor model using MC38^HER2^ cells. We devided the mice into four groups randomly and treated with PBS (control group), RC48, anti-PD-1, or a combination of RC48 and anti-PD-1. Both RC48 and anti-PD-1 alone had an inhibitory effect on tumor growth, while the combination therapy enhanced the effect without significant reduction in body weight, suggesting that RC48 can sensitize immunotherapy (Fig. 5A–E). The killing effect of Disitamab alone on tumors is not as significant as that of RC48 (Supplementary Fig E–I). To assess the long-term therapeutic effect of the combination therapy in CRC, the survival of tumor-bearing mice was monitored. Our results showed that the overall survival of mice in the combination therapy group was significantly prolonged comparing to PBS and RC48 groups (Fig. 5G). Our further investigation of the combination of RC48 and anti-PD-1 therapy in the murine model presented remarkable destruction of tumor cells as evidenced by H&E staining, which showed nuclear shrinkage and disordered arrangement. Results from immunohistochemistry showed a decrease in the expression of PCNA, indicating a decrease in proliferative capacity. The level of apoptosis in the combination group also increased according to the results of TUNEL staining (Fig. 5F). The same results were also confirmed in the CT26^HER2^ syngeneic model (Fig. 5H–M).

**Fig. 6:**
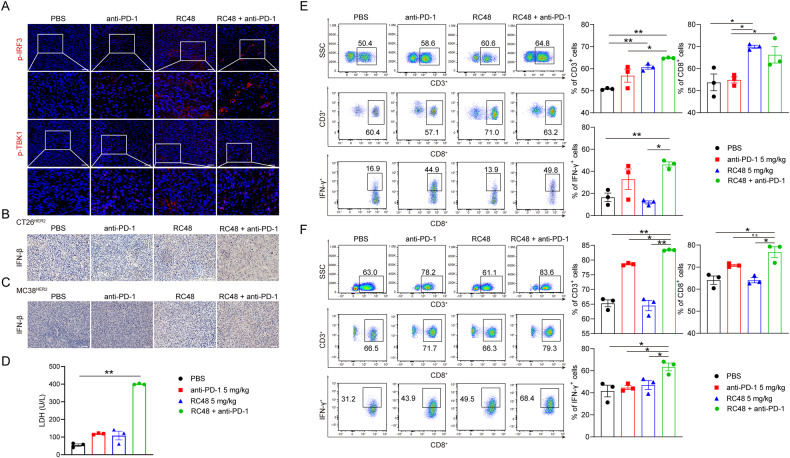
RC48 sensitized anti-PD-1 therapy via activating the cGAS-STING pathway and promoting immune cell infiltration. **A** Immunofluorescence staining for p-TBK1 and p-IRF3 in tumor tissue in C57BL/6. **B**, **C** Immunohistochemical detection of IFN-β secretion in tumor tissue in BALB/c and C57BL/6. **D** LDH release assay was performed using Tils achieved from the syngeneic model of CT26^HER2^ as described in material and methods. **E**, **F** Examination of CD3^+^, CD8^+^, IFN-γ secretion in the tumor by flow cytometry in BALB/c and C57BL/6. *P < 0.05, **P < 0.01 versus as indicated. n.s. not significant. Scale bar, 100 μm.

The above results support the therapeutic effect of RC48. We then sought to validate the mechanism by which RC48 triggers immunotherapy sensitization. Immunofluorescence staining showed a significant increase in the presence of p-TBK1 and p-IRF3 in both the RC48 and the combination groups (Fig. 6A). Also, a significant increase in IFN-β could be observed in the same groups (Fig. 6B, C). Despite the improved efficacy of anti-PD-1 therapy, the PD-L1 levels on the surface of tumor cells did not change after the use of RC48, both in vitro and in vivo (Supplementary Fig. B, C). We then performed a series of experiments to examine the functions of immune cells and their relationship to the RC48 treatment. The result of the LDH release assay demonstrated that the lymphocytes achieved from the combination group had higher cytotoxic activity (Fig. 6D). Flow cytometry showed a significant increase in the amount of CD3^+^, CD3^+^CD8^+^, and CD8^+^ IFN-γ^+^ cells in CT26^HER2^ tumor tissue (Fig. 6E), which was confirmed in the MC38^HER2^ syngeneic model (Fig. 6F). These results indicate that the sensitization of the immune system is not limited by microsatellite status of the cells, as both the MC38 and CT26 cell lines have different microsatellite stability (MSI for MC38 and MSS for CT26).

In conclusion, the results demonstrate that the combination of RC48 and anti-PD-1 therapy significantly inhibited tumor growth in the syngeneic model of CRC through both the direct killing of tumor cells and the promotion of anti-tumor immunity.

## Discussion

The proposal of the concept, ADC, dates back to a century ago, when Nobel Prize winner immunologist Paul Ehrlich predicted the potential for a chemotherapy drug with precise targeting capability to deliver toxins to cancer cells. After a hundred years of technological advancements, ADC finally entered the world of antitumor drugs in the early 2000s [25]. How groundbreaking guided missile is to plain firebomb, ADC is distinct from chemotherapy drugs, as the combination of an antibody and a toxin makes the drugs complementary to each other. The targeting aspect of the antibody weakens the toxic effect of the toxin on healthy cells, allowing for the use of highly toxic drugs that were previously discarded due to side effects. For example, MMAE is the synthetic derivative of dolastatin whose killing effect was not ideal at the dose that the human can tolerate (450 μg/m^2^, equal to 12 μg/kg in a 60 kg patient) [26]. ADC drugs, RC48, have brought new opportunities to it that the dose can reach 2.5 mg/kg (equal to 50 μg/kg MMAE) [27]. Our research found that only HER2-positive cells could be targeted and killed by RC48. This increases the tolerated dose and widens the range of applications. In addition, the limitation of mAbs caused by tumor heterogeneity is also overcome by the bystander effect of ADC drugs where toxins that have fallen off from the drug after binding to a tumor membrane receptor can directly destroy cells nearby, even those without the particular receptor.

RC48 has produced impressive results in clinical trials, including similar overall response rates (ORRs) in subgroups of patients with both low and high HER2 expression, a result that was rarely seen in classical HER2 mAbs. It correlates with the high affinity of Disitamab to HER2 compared to trastuzumab [24]. However, there are concerns about the failure of paclitaxel in colorectal cancer clinical trials, as paclitaxel resistance in colon cancer is related to drug transport by p-glycoprotein, reducing the concentration of the drug in cells. Our research showed that MMAE is much more toxic than paclitaxel at the same concentration, and we suspect that this increased toxicity counteracts the transport of p-glycoprotein, allowing MMAE to still produce a strong lethal effect even at low concentrations.

Immunotherapy, represented by anti-programmed death-1 (PD-1), is rapidly advancing in the field of cancer treatment. However, in colorectal cancer, only patients with high levels of microsatellite instability (MSI-H) can benefit from it, making up only 5% of cases. To rescue the remaining 95% of patients, various attempts to sensitize immunotherapy in non-MSI-H patients have been done, such as the use of anti-angiogenic agents to increase the infiltration of TILs or using chemotherapy and radiotherapy to induce immunogenic cell death (ICD) and activate DC and CD8^+^ cells [28]. Besides, the impact of microbiota on immune function is also a hot topic these years. For example, the use of antibiotics will weaken immunotherapy and the intervention of intestinal flora in patients who are not sensitive to immunotherapy may improve the efficacy [29]. Despite these efforts, much work needs to be done to improve immunotherapy response in microsatellite stable (MSS) patients. The effectiveness of immunotherapy is closely related to antitumor immune function and the cGAS-STING pathway plays a crucial role in this function. STING (stimulator of interferon genes) is an important adaptor protein in the production of type I interferons, with a transmembrane domain that localizes itself to the endoplasmic reticulum (ER) and a cytoplasmic domain that is involved in STING activation and interactive to downstream proteins [30, 31]. The cGAS-STING pathway is critical in both antitumor and antiviral immunity [32]. This study investigated the individual effects of the two components of RC48-ADC: MMAE and Disitamab. The toxicity of MMAE as a microtubulin inhibitor in colon cancer was confirmed, but our initial hypothesis about its relationship with the cGAS-STING pathway was not supported by the negative results. However, we made an unexpected discovery of Disitamab’s role in the cGAS-STING pathway. In conclusion, the killing effect of RC48 on colon cancer cells was triggered by its two components: On the one hand, MMAE, a tubulin inhibitor, arrested the cell cycle in G2/M phase; On the other hand, Disitamab, a HER2 antibody, activates the cGAS-STING signaling pathway and increases IFN-β secretion, which can promote immune cell infiltration, enhance anti-tumor immunity, and improve the cytotoxic of tumor-infiltrating lymphocytes.

**Fig. 7:**
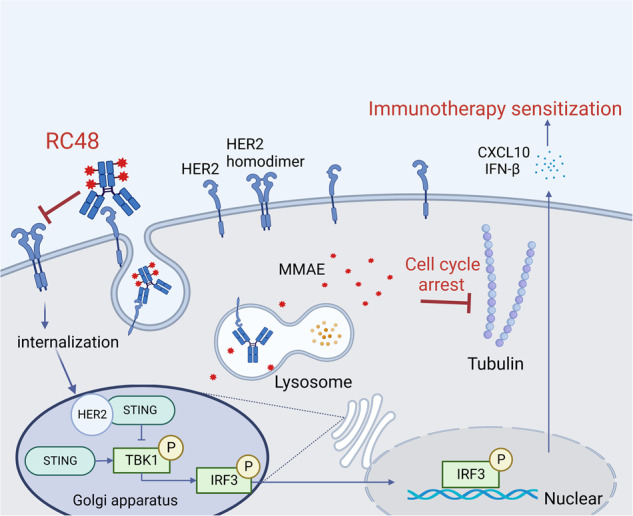
The graphic illustration of the mechanism for RC48. RC48 exhibits the capability to bind to HER2 receptors and undergo internalization, subsequently releasing the toxin MMAE. MMAE inhibits tubulin, leading to the disruption of the cell cycle and resulting in the efficient elimination of tumor cells. Moreover, through its interaction with HER2, RC48 can alleviate the inhibition induced by internalized HER2 on the STING pathway and its downstream signaling. These combined mechanisms enable RC48 to effectively impede the proliferation of HER2-positive tumors while also enhancing their sensitivity to immunotherapy.

Colon cancer is a highly prevalent tumor type with limited options for third-line treatments. This study investigated the efficacy of RC48 in colon cancer, establishing a solid experimental groundwork for future clinical research (Fig. 7). The findings indicate two potential benefits: Firstly, the tubulin inhibitor MMAE, although unconventional for colon cancer, shows promise in effectively treating patients who have developed resistance to conventional drugs. Secondly, the drug’s immunotherapy sensitizing effect discovered in this study presents a potential opportunity to rescue patients who exhibit no response to immunotherapy. Based on this, a single-arm, open-label, multiple centers, prospective study of Disitamab Vedotin combined with Tislelizumab in HER2-positive advanced colorectal cancer failed at least two lines of systemic treatment are ongoing in our hospital (NCT05493683). It is hoped that RC48 will help to overcome the challenges of immunotherapy in CRC patients.

## Materials and methods

### Chemicals and reagents

RC48 and Disitamab were provided by RemeGen Co., Ltd. Monomethyl auristatin E (MMAE, 474645-27-7) was from Topscience Co. Ltd. Irinotecan (CPT-11, I1406), were purchased from Sigma-Aldrich LLC. Anti-mouse PD-1 antibody (clone RMP1-14) was purchased from Bio X Cell.

### Cell culture

The murine colon carcinoma cell lines MC38, CT26, the human colon carcinoma cell lines HT29, HCT116, and the human breast carcinoma cell lines 468, ZR-75-1 was derived from the Type Culture Collection of the Chinese Academy of Sciences. CT26 and 468 cells were cultured in RPMI 1640 medium, and the other four cell lines were cultured in Dulbecco’s modified Eagle’s medium (DMEM).

### Cell viability

Two thousand cells were plated into 96-well plates and incubated with various concentrations of drugs. At the indicated time points, cell viability was examined by the MTT assay. MTT (4 mg/ml, 20 µl/well) was added. After 4 h of additional incubation, the plates were centrifugated at 1500 rpm for 5 min to remove the upper layer. DMSO was added into each well before detecting absorption values at 570 nm.

### Animals

Eight-week-old female C57BL/6, BALB/c mice were purchased from Tande Biotechnology. They were housed at 21 ± 2 °C and kept on a 12-h light/dark rhythm as previously described [33]. Animal welfare and experimental procedures were conducted according to the Guide for the Care and Use of Laboratory Animals from the National Institutes of Health and the ethical regulations of Nanjing Medical University (Animal ethical approval number: IACUC-2210010).

### Syngeneic model

MC38^HER2^ or CT26^HER2^ cells (1 × 10^6^ in 100 μl PBS) were inoculated into the right flank of the C57BL/6 or BALB/c mice subcutaneously. The mice were divided into different groups after the tumor volume reached 50–100 mm^3^ without randomization. They received intravenous injections (i.v.) of RC48, Disitamab or PBS, intraperitoneal injection (i.p.) of PD-1 antibody, or PBS without blinding. Tumors were collected 2 or 3 days after the last intervention. The tumor volume was measured by assessing the length and width of the tumor using a digital caliper and the body weight was recorded every 2 days as previously described [33]. The volume was calculated by the formula: Volume = long axis × short axis^2^/2. The tumor weight was measured after tumor collection.

### Isolation of tumor-infiltrating lymphocytes (TILs)

The tumor tissue was processed by a murine tumor dissociation kit from Miltenyi Biotec (130-0960-730, Bergisch Gladbach), which involved both mechanical and enzymatic dissociation. After filtering the single-cell suspension through a 70 μm mesh, the Tumor Infiltrating Lymphocytes (TILs) were then obtained by separating the cells using a Percoll gradient (40–70%) and centrifuging them at 700 × *g* for 20 min. The resulting cell fraction was resuspended in RMPI 1640 medium with 10% Fetal Bovine Serum (FBS) for further use.

### Flow cytometry

Anti-human HER2-APC (324407, Biolegend) was used to detect the HER2 expression on the cell membrane. The TILs were stained with the following antibodies: Zombie NIRTM (423105, Biolegend) for dead cell preclusion, CD45-BV510 (103138, Biolegend), CD3-APC (100236, Biolegend), CD4-FITC (100406, Biolegend), CD8-BV421 (100738, Biolegend), IFN-γRα-PE (308606, Biolegend).

### IHC and TUNEL assays

The tumor sections were first embedded in 100% xylene-dewaxed paraffin and then rehydrated with gradient ethanol. After the endogenous peroxidase was exhausted and the antigens were blocked, IHC staining was carried out by an Immunohistochemical Detection kit (Proteintech). TUNEL staining was performed using the Cell Apoptosis Detection Kit (Trevigen). The photograph was obtained with a light microscope (Olympus). Anti-PCNA (GB11010) was purchased from Servicebio (Wuhan, China).

### Lentivirus and transfections

A lentivirus overexpressing HER2 was generated by lentiviral transduction using the pSLenti-EF1-mCherry-P2A-Puro-CMV-ERBB2-3xFLAG-WPRE (OBIO Technology (Shanghai) Corp., LTD.). A control lentivirus without the HER2 construct (LV-control) was also created using an empty vector. The transfection process was carried out according to the manufacturer’s instructions using polybrene as the transfection agent.

### Real-time RT-PCR analysis

The extraction of the total RNA was performed by TRIzol reagent (TaKaRa) and the FastQuant RT Kit (Tiangen) was used to reverse transcribe. Then the SYBR Green Kit (Invitrogen) was used in qPCR analysis and it was quantified by the Real-Time PCR Detection system (Bio-rad Laboratories). The relative mRNA levels normalized according to the level of β-actin. Primer sequences were as follows: β-actin, forward 5′-CGCGAGAGAAGATGACCCAGATC-3′ and reverse 5′-GCCAGAGGCGTACAGGGATA-3′. IFN-β, forward 5′-ACGCCGCATTGACCATCTAT-3′ and reverse 5′-GTCTCATTCCAGCCAGTGCT-3′. CXCL10, forward 5′-AGCAGAGGAACCTCCAGTCT-3′ and reverse 5′-AGGTACTCCTTGAATGCCACT-3′. HER2, forward 5′-TGGCCTGTGCCCACTATAAG-3′ and reverse 5′-AGGAGAGGTCAGGTTTCACAC-3′. CCL5, forward 5′-CTGCTTTGCCTACATTGCCC-3′ and reverse 5′-TCGGGTGACAAAGACGACTG-3′.

### Western blot analysis

Cells were digested in lysis buffer (Beyotime) on ice. SDS-PAGE was used to separate equivalent loading proteins and they were transferred onto polyvinylidene difluoride membranes (Millipore). Next, they were blocked with 3% BSA or 5% nonfat milk before reaction with antibodies at 4 °C overnight and then with secondary antibodies for 2 h. Finally, immunoreactive bands were detected using an ECL kit (Tanon). All the procedures above were as previously described [33]. Anti-HER2 antibody (ab134182) was from Abcam plc. Anti-STING (13647), anti-pTBK1 (5483), and anti-p-IRF3 (29047) were purchased from Cell Signaling Technology. Anti-TBK1 (28397-1-AP) and anti-IRF3 (11312-1-AP) were purchased from Proteintech. Anti-β-Actin (M20010) and anti-GAPDH (M20005) were purchased from Abmart. Anti-IFN-β (YT0365) was purchased from Immunoway. Anti-PD-L1 (A1645) was purchased from Abclonal.

### Immunofluorescence

The fixation, permeabilization, and blocking were respectively performed by 4% formaldehyde, 0.5% TritonX-100 (Beyotime), and 5% BSA. Samples were reacted with antibodies at 4 °C overnight and then with secondary antibodies for 2 h. After staining with DAPI, they were imaged with fluorescence microscopy (Olympus). All the procedures above were as previously described [20].

### Clonogenic assay

Preconditioned cells were seeded in six-well plates and allowed to adhere for 6 h, treated with different drug concentrations for 48 h. Then the plates were replaced with normal medium, and cells were allowed to incubate for 10 days. Plates were then stained with crystal violet, and colonies consisting of 50 or more cells were manually counted.

### Coimmunoprecipitation assay

Cells were treated with RIPA buffer. The supernatant of whole cell lysate (1 mg) and 1 mg antibody were reacted overnight at 4 °C, and then the immune complex was incubated with protein A/G beads (Santa Cruz Biotechnology) for 4 h. After washing five times in lysis buffer, the immune complex was analyzed by Western blot as previously described [20].

### Cell-cycle analyses

The Cell Cycle Detection Kit (KGA512, KeyGEN) was used to detect the cell cycle. FlowJo software was used to analyze the data.

### Fluorescence in situ hybridization (FISH) assays

Samples were fixed in 4% formaldehyde for 15 min and then washed with PBS. Pepsin (1% in 10 mmol/l HCl) was added to the sample, followed by continuous dehydration with ethanol. The dried samples were mixed with 40 nmol/l of the FISH probe in a hybridization buffer and incubated at 80 °C for 2 min. After staying at 55 °C for 2 h, the slides were washed and dehydrated, and finally observed after stained with DAPI and washed with PBS.

### LDH release assay

The LDH release detection kit (Jiancheng Bioengineering Institute) was used according to the manufacturer’s protocol. Tumor-infiltrating lymphocytes prestimulated by IL-2 for 48 h and tumor cells were plated in 96-well plates (1 × 10^5^ cells/100 μl per well, effector-target ratio: 50:1). 48 h later, the supernatants were harvested to measure the release of LDH from the cells. The experiments were performed in triplicate.

### Statistical analysis

Statistical analyses were performed using the GraphPad Prism software (version 8). All data were presented as the mean ± SEM. One-way ANOVA was used to analyze statistically significant differences between multiple-group comparisons. A two-tailed Student *t*-test was used to analyze statistically significant differences between the two groups as previously described [20]. *P* < 0.05 was considered statistically significant.

## Supplementary information

supplementary data

Original Data File

checklist

## Data Availability

All datasets generated and analyzed during this study are included in this published article and its Supplementary Information files. Additional data are available from the corresponding author on reasonable request.

## References

[1] Xia C, Dong X, Li H, Cao M, Sun D, He S (2022). Cancer statistics in China and United States, 2022: profiles, trends, and determinants. Chin Med J.

[2] Van Cutsem E, Cervantes A, Adam R, Sobrero A, Van Krieken JH, Aderka D (2016). ESMO consensus guidelines for the management of patients with metastatic colorectal cancer. Ann Oncol.

[3] Oh DY, Bang YJ (2020). HER2-targeted therapies - a role beyond breast cancer. Nat Rev Clin Oncol.

[4] Kumagai S, Koyama S, Nishikawa H (2021). Antitumour immunity regulated by aberrant ERBB family signalling. Nat Rev Cancer.

[5] Ni S, Wang X, Chang J, Sun H, Weng W, Wang X (2022). Human epidermal growth factor receptor 2 overexpression and amplification in patients with colorectal cancer: a large-scale retrospective study in Chinese population. Front Oncol.

[6] Essapen S, Thomas H, Green M, De Vries C, Cook MG, Marks C (2004). The expression and prognostic significance of HER-2 in colorectal cancer and its relationship with clinicopathological parameters. Int J Oncol.

[7] Ivanova M, Venetis K, Guerini-Rocco E, Bottiglieri L, Mastropasqua MG, Garrone O (2022). HER2 in metastatic colorectal cancer: pathology, somatic alterations, and perspectives for novel therapeutic schemes. Life.

[8] Torres-Jimenez J, Esteban-Villarrubia J, Ferreiro-Monteagudo R (2022). Precision medicine in metastatic colorectal cancer: targeting ERBB2 (HER-2) oncogene. Cancers.

[9] Karan C, Tan E, Sarfraz H, Knepper TC, Walko CM, Felder S (2022). Human epidermal growth factor receptor 2-targeting approaches for colorectal cancer: clinical implications of novel treatments and future therapeutic avenues. JCO Oncol Pr.

[10] Chang J, Xu M, Wang C, Huang D, Zhang Z, Chen Z (2022). Dual HER2 targeted therapy with pyrotinib and trastuzumab in refractory HER2 positive metastatic colorectal cancer: a result from HER2-FUSCC-G study. Clin Colorectal Cancer.

[11] Bertotti A, Migliardi G, Galimi F, Sassi F, Torti D, Isella C (2011). A molecularly annotated platform of patient-derived xenografts (“xenopatients”) identifies HER2 as an effective therapeutic target in cetuximab-resistant colorectal cancer. Cancer Discov.

[12] Meric-Bernstam F, Hurwitz H, Raghav KPS, McWilliams RR, Fakih M, VanderWalde A (2019). Pertuzumab plus trastuzumab for HER2-amplified metastatic colorectal cancer (MyPathway): an updated report from a multicentre, open-label, phase 2a, multiple basket study. Lancet Oncol.

[13] Sartore-Bianchi A, Trusolino L, Martino C, Bencardino K, Lonardi S, Bergamo F (2016). Dual-targeted therapy with trastuzumab and lapatinib in treatment-refractory, KRAS codon 12/13 wild-type, HER2-positive metastatic colorectal cancer (HERACLES): a proof-of-concept, multicentre, open-label, phase 2 trial. Lancet Oncol.

[14] Tarantino P, Carmagnani Pestana R, Corti C, Modi S, Bardia A, Tolaney SM (2022). Antibody-drug conjugates: Smart chemotherapy delivery across tumor histologies. CA Cancer J Clin.

[15] Shi F, Liu Y, Zhou X, Shen P, Xue R, Zhang M (2022). Disitamab vedotin: a novel antibody-drug conjugates for cancer therapy. Drug Deliv.

[16] Peng Z, Liu T, Wei J, Wang A, He Y, Yang L (2021). Efficacy and safety of a novel anti-HER2 therapeutic antibody RC48 in patients with HER2-overexpressing, locally advanced or metastatic gastric or gastroesophageal junction cancer: a single-arm phase II study. Cancer Commun.

[17] Sheng X, Yan X, Wang L, Shi Y, Yao X, Luo H (2021). Open-label, multicenter, phase II study of RC48-ADC, a HER2-targeting antibody-drug conjugate, in patients with locally advanced or metastatic urothelial carcinoma. Clin Cancer Res.

[18] Xu Y, Wang Y, Gong J, Zhang X, Peng Z, Sheng X (2021). Phase I study of the recombinant humanized anti-HER2 monoclonal antibody-MMAE conjugate RC48-ADC in patients with HER2-positive advanced solid tumors. Gastric Cancer.

[19] Hu Y, Manasrah BK, McGregor SM, Lera RF, Norman RX, Tucker JB (2021). Paclitaxel induces micronucleation and activates pro-inflammatory cGAS-STING signaling in triple-negative breast cancer. Mol Cancer Ther.

[20] Wei B, Xu L, Guo W, Wang Y, Wu J, Li X (2021). SHP2-mediated inhibition of DNA repair contributes to cGAS-STING activation and chemotherapeutic sensitivity in colon cancer. Cancer Res.

[21] Wang Y, Wei B, Wang D, Wu J, Gao J, Zhong H (2022). DNA damage repair promotion in colonic epithelial cells by andrographolide downregulated cGAS‒STING pathway activation and contributed to the relief of CPT-11-induced intestinal mucositis. Acta Pharmaceutica Sin B.

[22] Wu S, Zhang Q, Zhang F, Meng F, Liu S, Zhou R (2019). HER2 recruits AKT1 to disrupt STING signalling and suppress antiviral defence and antitumour immunity. Nat Cell Biol.

[23] Janjigian YY, Kawazoe A, Yanez P, Li N, Lonardi S, Kolesnik O (2021). The KEYNOTE-811 trial of dual PD-1 and HER2 blockade in HER2-positive gastric cancer. Nature.

[24] Huang L, Wang R, Xie K, Zhang J, Tao F, Pi C (2021). A HER2 target antibody drug conjugate combined with anti-PD-(L)1 treatment eliminates hHER2+ tumors in hPD-1 transgenic mouse model and contributes immune memory formation. Breast Cancer Res Treat.

[25] Chau CH, Steeg PS, Figg WD (2019). Antibody-drug conjugates for cancer. Lancet.

[26] Saad ED, Kraut EH, Hoff PM, Moore DF, Jones D, Pazdur R (2002). Phase II study of dolastatin-10 as first-line treatment for advanced colorectal cancer. Am J Clin Oncol.

[27] Jiang J, Li S, Shan X, Wang L, Ma J, Huang M (2020). Preclinical safety profile of disitamab vedotin: a novel anti-HER2 antibody conjugated with MMAE. Toxicol Lett.

[28] Ganesh K, Stadler ZK, Cercek A, Mendelsohn RB, Shia J, Segal NH (2019). Immunotherapy in colorectal cancer: rationale, challenges and potential. Nat Rev Gastroenterol Hepatol.

[29] Agagunduz D, Cocozza E, Cemali O, Bayazit AD, Nani MF, Cerqua I (2023). Understanding the role of the gut microbiome in gastrointestinal cancer: a review. Front Pharm.

[30] Kwon J, Bakhoum SF (2020). The cytosolic DNA-sensing cGAS-STING pathway in cancer. Cancer Discov.

[31] Ding C, Song Z, Shen A, Chen T, Zhang A (2020). Small molecules targeting the innate immune cGAS-STING-TBK1 signaling pathway. ACTA PHARMACEUTICA SINICA B.

[32] Zhang X, Bai XC, Chen ZJ (2020). Structures and Mechanisms in the cGAS-STING Innate. Immun Pathw Immun.

[33] Li X, Lu M, Yuan M, Ye J, Zhang W, Xu L (2022). CXCL10-armed oncolytic adenovirus promotes tumor-infiltrating T-cell chemotaxis to enhance anti-PD-1 therapy. Oncoimmunology.

