# Supplementary material for: A HER2-targeting antibody-MMAE conjugate RC48 sensitizes immunotherapy in HER2-positive colon cancer by triggering the cGAS-STING pathway
Source: Cell Death Dis. 2023 Aug 24;14(8):550. doi: 10.1038/s41419-023-06073-8 (PMC10449775; doi:10.1038/s41419-023-06073-8)
Supplement: Supplementary file 1 — supplementary data [file 41419_2023_6073_MOESM1_ESM.pdf]

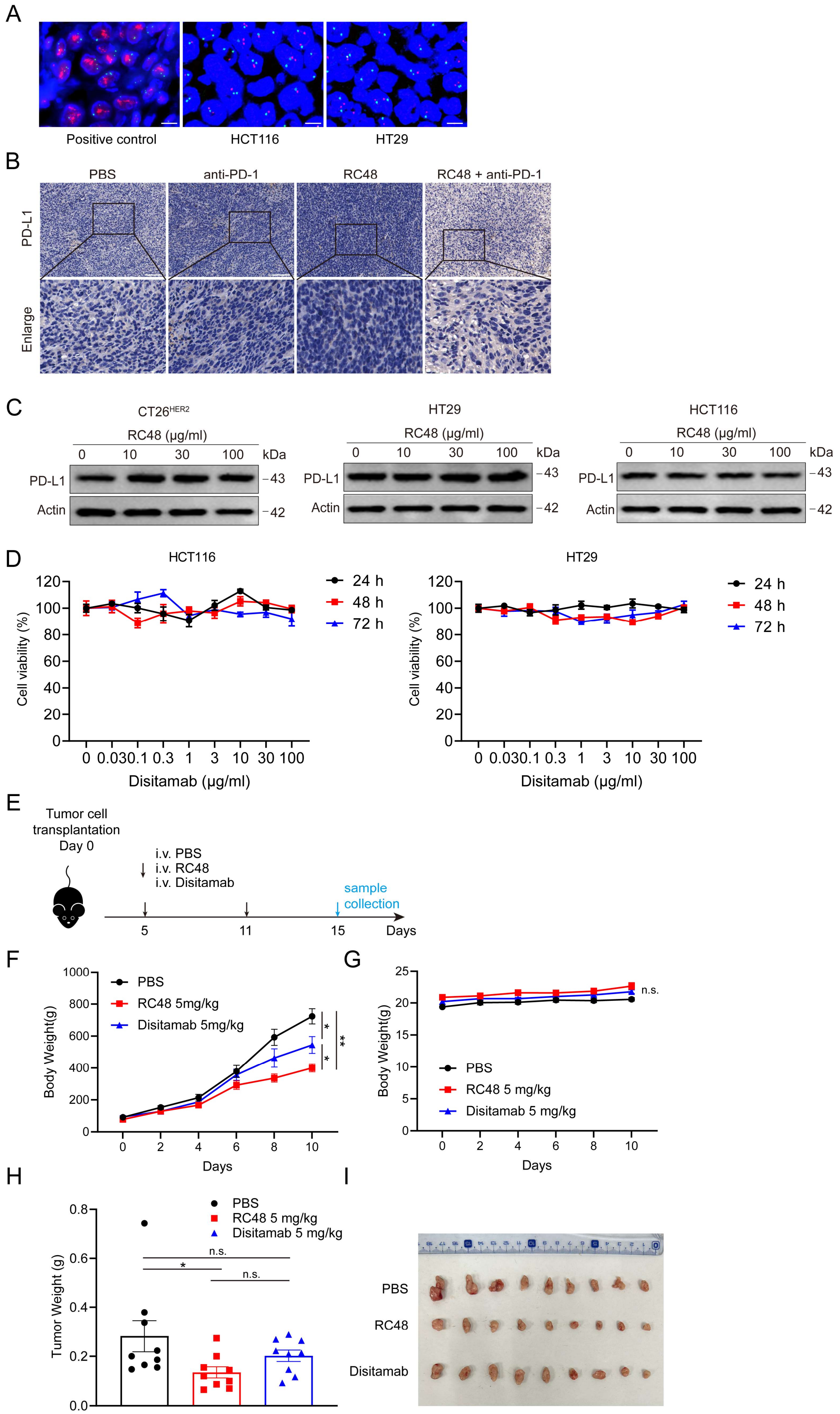

A
Positive control
HCT116
HT29
B
PBS
anti-PD-1
RC48
RC48 + anti-PD-1
PD-L1
Enlarge
C
CT26HER2
RC48 (μg/ml)
0 10 30 100 kDa
PD-L1 -43
Actin -42
HT29
RC48 (μg/ml)
0 10 30 100 kDa
PD-L1 -43
Actin -42
HCT116
RC48 (μg/ml)
0 10 30 100 kDa
PD-L1 -43
Actin -42
D
HCT116
HT29
24 h
48 h
72 h
Cell viability (%)
0 0.03 0.1 0.3 1 3 10 30 100
Disitamab (μg/ml)
E
Tumor cell transplantation Day 0
i.v. PBS
i.v. RC48
i.v. Disitamab
sample collection
5
11
15
Days
F
PBS
RC48 5mg/kg
Disitamab 5mg/kg
Body Weight(g)
Days
G
n.s.
PBS
RC48 5 mg/kg
Disitamab 5 mg/kg
Body Weight(g)
Days
H
PBS
RC48 5 mg/kg
Disitamab 5 mg/kg
Tumor Weight (g)
n.s.
n.s.
I
PBS
RC48
Disitamab

Supplementary Fig 1. (A) FISH assay on HCT116 and HT29, and a sample from a HER2 amplified patient as a positive control. Scale bar, 10 μm. (B) Immunohistochemical detection of IFN-β secretion in tumor tissue in C57BL/6. Scale bar, 100 μm. (C) $CT26^{HER2}$, HT29, HCT116 cells were treated with different concentrations of RC48 (0, 10, 30, 100 μg/ml) for 48 h, and the expression of PD-L1 protein was detected by immunoblotting. (D) *In vitro* growth inhibition assay for HCT116 and HT29 cells with Disitamab for 24, 48, 72 hours. (E) $MC38^{HER2}$ (1×10^6) cells were inoculated in the C57BL/6 mice. (F, G) Tumor volume and body weight were measured every two days. (H, I) Solid tumors were separated and weighed after the mice were sacrificed. The data represent the mean ±SEM of 8 mice per group. * $P < 0.05$, ** $P < 0.01$ versus as indicated. n.s., not significant.
