# Supplementary figures and images for: A HER2-targeting antibody-MMAE conjugate RC48 sensitizes immunotherapy in HER2-positive colon cancer by triggering the cGAS-STING pathway
Source: Cell Death Dis. 2023 Aug 24;14(8):550. doi: 10.1038/s41419-023-06073-8 (PMC10449775; doi:10.1038/s41419-023-06073-8)

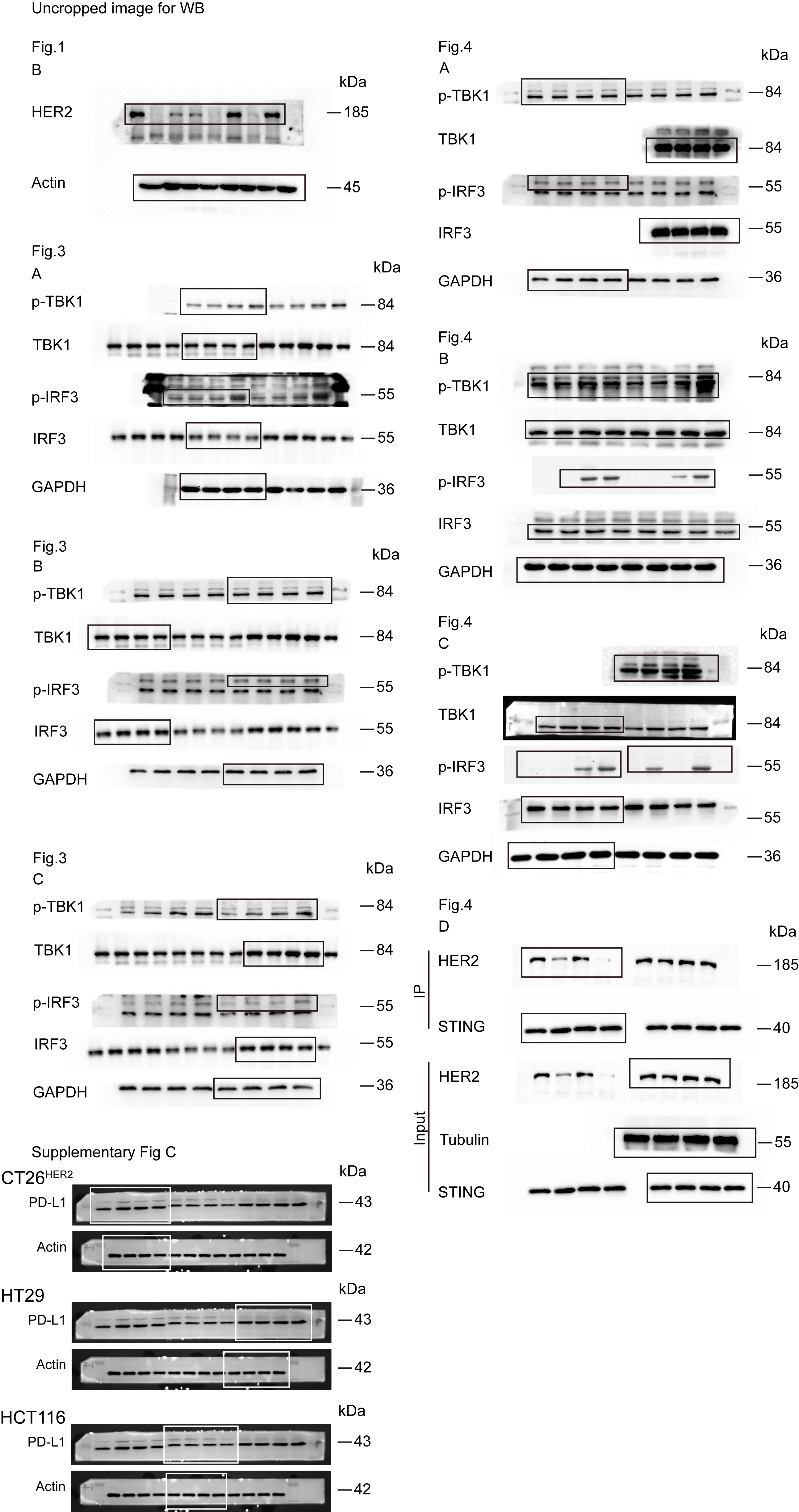

Supplement: Supplementary file 2 — Original Data File [file 41419_2023_6073_MOESM2_ESM.tif]
